# Separating the microbiome from the hyperbolome

**DOI:** 10.1186/s13073-015-0143-5

**Published:** 2015-02-24

**Authors:** Fergus Shanahan

**Affiliations:** Department of Medicine, Alimentary Pharmabiotic Centre, University College Cork, National University of Ireland, Cork, Republic of Ireland

## Abstract

Microbiome-based therapies are moving quickly towards the clinic, with successes including fecal microbial transplants for recurring *Clostridium difficile*, hints of new antibiotics to come, and possible new microbial biomarkers for common complex diseases. Can the microbiome live up to its hype?

“Let us leave theories there and return to here’s here.”

- James Joyce, *Finnegans Wake* [1]

It has become fashionable to advise against overstating the role of the microbiome in health and disease and to warn of the danger of mistaking correlation for causation [2]. Hype and hyperbole hindered science and medicine long before any resurgence of interest in the microbiome, and self-evident nuggets of received wisdom and skepticism should not distract from the success stories and lessons already generated by this field. Indeed, few areas in science have been translated as quickly to therapeutic medicine. Advances range from therapeutic modification of the microbiota with probiotics, antibiotics or fecal microbial transplantation (FMT), to the use of microbial compositional changes as biomarkers of disease risk and the exploration of the microbiota as a repository for natural agents that could be harnessed as therapeutics [3].

## From microbes to therapies

Arguably, the most important and therapeutically significant discovery that has arisen from studying the microbiota is the link between *Helicobacter pylori* and peptic ulcer disease, gastric cancer and some forms of gastric lymphoma [4]. This was the result of fresh thinking across artificial boundaries of knowledge and exposed the folly of traditional ‘risk factor epidemiology’, which had missed a transmissible agent in peptic ulcer disease for many decades. The *H. pylori* story is not a simple tale of infection and host response, ending with elimination of the organism. The truth is more complex, and included the protective effects of *H. pylori* against reflux and cancer of the gastro-esophageal junction. This much we know; but the possibility that loss of *H. pylori* and other ancient microbes in a modern world might predispose to metabolic, immune and allergic disorders is too important to ignore [4]. The most important lesson from this discovery was that there are some diseases, seemingly complex and heterogeneous, that cannot be solved by research focused only on the host, without due regard for the microbial environment. It is no overstatement to predict that other complex disorders, such as colon cancer and perhaps some forms of inflammatory bowel disease, might likewise have a microbial basis, although not necessarily with a one-microbe-one-disease relationship; consortia rather than individual microbes may affect disease risk depending on host susceptibility.

The best evidence for the therapeutic benefits of manipulating the microbiota is ancient - FMT is an old technique that has been rediscovered and now widely accepted for its consistent efficacy against recurring *Clostridium difficile*-associated disease [5]. Regrettably, this striking success was accompanied by overblown expectations that the same treatment might work in a diversity of other conditions, such as irritable bowel syndrome, inflammatory bowel disease or even extra-intestinal disorders. While science continues this microbiological experiment to determine the minimum necessary microbiota for a therapeutic effect and the mechanisms underlying this, and regulatory agencies struggle to catch up, FMT is intriguing to many patients, raising concerns about unsupervised, self-administration. Obvious safety concerns include transmission of an infection, which is preventable for known pathogens, and bacterial translocation in recipients with an impaired mucosal barrier who are challenged with the intake of a huge bacterial load. In addition, one of the lessons from FMT in experimental animals is that immunologic, behavioral and metabolic phenotypes can be transferred from donor to recipient. If this extrapolates to humans, it introduces a risk of long-term adverse consequences, particularly for young recipients. Since FMT in humans has been shown to transfer an improved metabolic phenotype from lean donors to less healthy recipients, the reverse could also be true. This implies that donor selection for FMT should not be based solely on exclusion of transmissible infections. Meanwhile, the availability of an artificial stool, populated with a diversity of known, well-characterized organisms, would circumvent most of these concerns, and is in development by several research groups.

## Harnessing microbial metabolism

New microbiome-informed therapies can be anticipated as science progresses beyond the identification of microbes linked with health or disease toward an exploration of their function. Harnessing microbial metabolism in the treatment of disease is an old strategy. For almost a century, clinicians have relied on colonic microbial enzymes to release the active agent aminosalicylate from its parent pro-drug, sulfasalazine, to treat colitis. More recently, the specific inhibition of microbial enzymes has been a therapeutic landmark in cancer chemotherapy [6]. Thus, the toxicity of irinotecan (formerly known as CPT-11), used in the treatment of colorectal and other cancers, can be attenuated by inhibition of bacterial glucuronidase. Irinotecan is activated *in vivo* after parenteral administration, metabolized by glucuronidation in the liver before excretion in bile, and then reactivated by bacterial glucuronidase in the bowel, where it may cause dose-limiting diarrhea. Drugs designed to selectively inhibit the bacterial but not the mammalian glucuronidase conferred protection against irinotecan toxicity without any antimicrobial effect. The prospects for other bacterial enzymes that may be tractable for therapeutic benefit are extensive and include bile salt hydrolases, which have been manipulated experimentally to favorably influence lipid metabolism, weight gain and cholesterol levels in the host [7].

Of course, the most direct way to alter the composition and metabolism of the microbiota is through dietary changes. Anyone who has ever changed a diaper from a breastfed baby who is being weaned to formula feed will be aware of the influence of diet on the fecal microbiota. Microbial diversity is a biomarker of a healthy enteric ecosystem. In a study of the elderly, diminished microbial diversity followed a reduction in dietary diversity, and the decrease in microbial diversity correlated with poor health [8]. While some might debate the directional nature of this correlation [2], the critical conclusion is that adequate nutrition is no longer a matter of quantity and quality, it also requires diversity. In addition to the importance of dietary diversity, microbiome science is poised to reveal a mechanistic basis for the health benefits of specific diets, such as high-fiber and elemental diets, because their beneficial effects appear to be due, in part, to an alteration in bacterial composition and metabolism.

Microbiome science has also cast a fresh perspective on the dual problems of diminished development of new antimicrobials by the pharmaceutical industry and increasing antibiotic resistance. First, the microbiota is an inner biomass from which new antimicrobial agents may be mined. Proof of principle has been established with the discovery of a peptide bacteriocin antibiotic with a high degree of specificity against *C. difficile* [9]. The microbiota may also be a convenient if untapped resource to be mined for therapeutically useful bacteriophages. Second, while campaigns for the more judicious use of antibiotics have had limited impact, the long-term consequences of disturbing the microbiota with antibiotics, particularly in early life, seems a compelling argument to change consumers’ attitudes and reduce unnecessary use of antibiotics. Evidence from human and animal studies suggests that exposure to antibiotics in early life, when the immune system is maturing, is a risk factor for later development of several chronic disorders, including inflammatory bowel disease, asthma, irritable bowel syndrome and metabolic disorders. The message seems clear: mind your microbes and they will mind you.

A recent conference organized by the National Institutes of Health reported on ‘amazing progress’ and ‘the potential for microbiome science to produce a revolution in human health’ [10]. Is this an overstatement? The advances already made suggest otherwise. Are those who dwell on false dawns and unfulfilled promises in science justified in their skepticism about the microbiome? James Joyce, a keen student of bowel function who died from the complications of *H. pylori*-related disease, said it best: “No assuredly, they are not justified, those gloompourers who grouse…” [1].

## Abbreviations

FMT: Fecal microbial transplantation
